# Transcatheter Aortic Valve Replacement for Bicuspid Aortic Valve Stenosis: A Practical Operative Overview

**DOI:** 10.1161/CIRCINTERVENTIONS.120.009827

**Published:** 2021-06-16

**Authors:** Giuseppe Tarantini, Tommaso Fabris

**Affiliations:** 1 Department of Cardiac, Thoracic, Vascular Sciences and Public Health, University of Padua, Italy.

**Keywords:** aortic stenosis, bicuspid aortic valve, multislice computed tomography, transcatheter heart valve, transcatheter aortic valve replacement

## Abstract

The bicuspid aortic valve (BAV) represents a complex anatomic scenario for transcatheter aortic valve replacement (TAVR) because of its unique technical challenges. As TAVR is moving towards younger and lower-risk populations, the proportion of BAV patients undergoing TAVR is expected to rise. Initial experiences of TAVR with first-generation transcatheter heart valves in high surgical risk patients with BAV stenosis showed higher rates of device failure and periprocedural complications as compared to tricuspid anatomy. The subsequent advances in imaging techniques and understanding of BAV anatomy, new iterations of transcatheter heart valves, and growing operators’ experience yielded better outcomes. However, in the lack of randomized trials and rigorous evidence, the field of TAVR in BAV has been driven by empirical observations, with wide variability in transcatheter heart valve sizing and implantation techniques across different centers and operators. Thus, in this review article, we provide a fully illustrated overview of operative periprocedural steps for TAVR in BAV stenosis, though recognizing that it still remains anecdotal.

## Abstract 2

Supplemental Digital Content is available in the text.

Bicuspid aortic valve (BAV) patients were systematically excluded from pivotal randomized trials comparing transcatheter aortic valve replacement (TAVR) and surgical AVR due to its unique unfavorable morphological features, for example, heavy and extreme asymmetry of valve calcifications, asymmetrical cusps’ size, commissural fusion (raphe), and associated aortopathy.^1^ Consequently, BAV was found in >20% of elderly (>80 years) and high-risk surgical AVR patients,^2^ who at present would have been referred to TAVR.

Early TAVR experiences with first-generation transcatheter heart valves (THVs) in high-risk bicuspid aortic stenosis (AS) patients were complicated by increased rates of paravalvular leak (PVL), new permanent pacemaker implantation, prosthesis embolization, aortic injury, and conversion to open surgery.^3,4^ Procedural outcomes improved with the refinement of imaging techniques, better understanding of BAV anatomy, new iterations of THVs, and growing experience of TAVR operators.^5–7^ However, in the absence of randomized trials and rigorous evidence, there is still no consensus on how to approach transcatheter treatment of bicuspid AS, especially regarding the optimal THV sizing (annular versus supraannular) and implantation strategy, leading to wide variability across different centers and operators.

This practical review aims to provide a fully illustrated overview of current periprocedural operative considerations for TAVR in BAV scenarios.

## Procedural Planning

**Figure 1.**
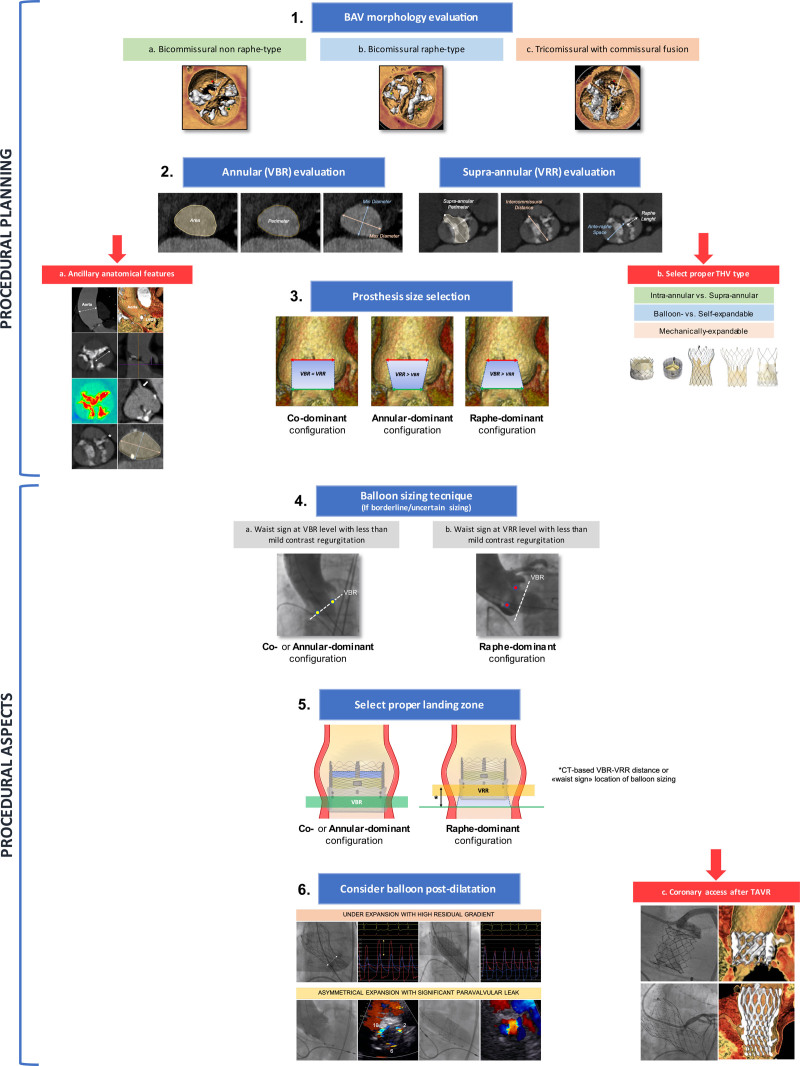
**Proposed operative sequence for transcatheter aortic valve replacement (TAVR) planning in bicuspid aortic valve (BAV).** VBR indicates virtual basal ring; VRR, virtual raphe ring; and THV, transcatheter heart valve.

A step-by-step approach based on both BAV anatomy, including the evaluation of BAV phenotypes, annular and supraannular (ie, at raphe level) dimensions, and ancillary anatomic features, is crucial to select the optimal THV size, type, and implantation height (Figure 1).

### BAV Morphology Characterization

**Figure 2.**
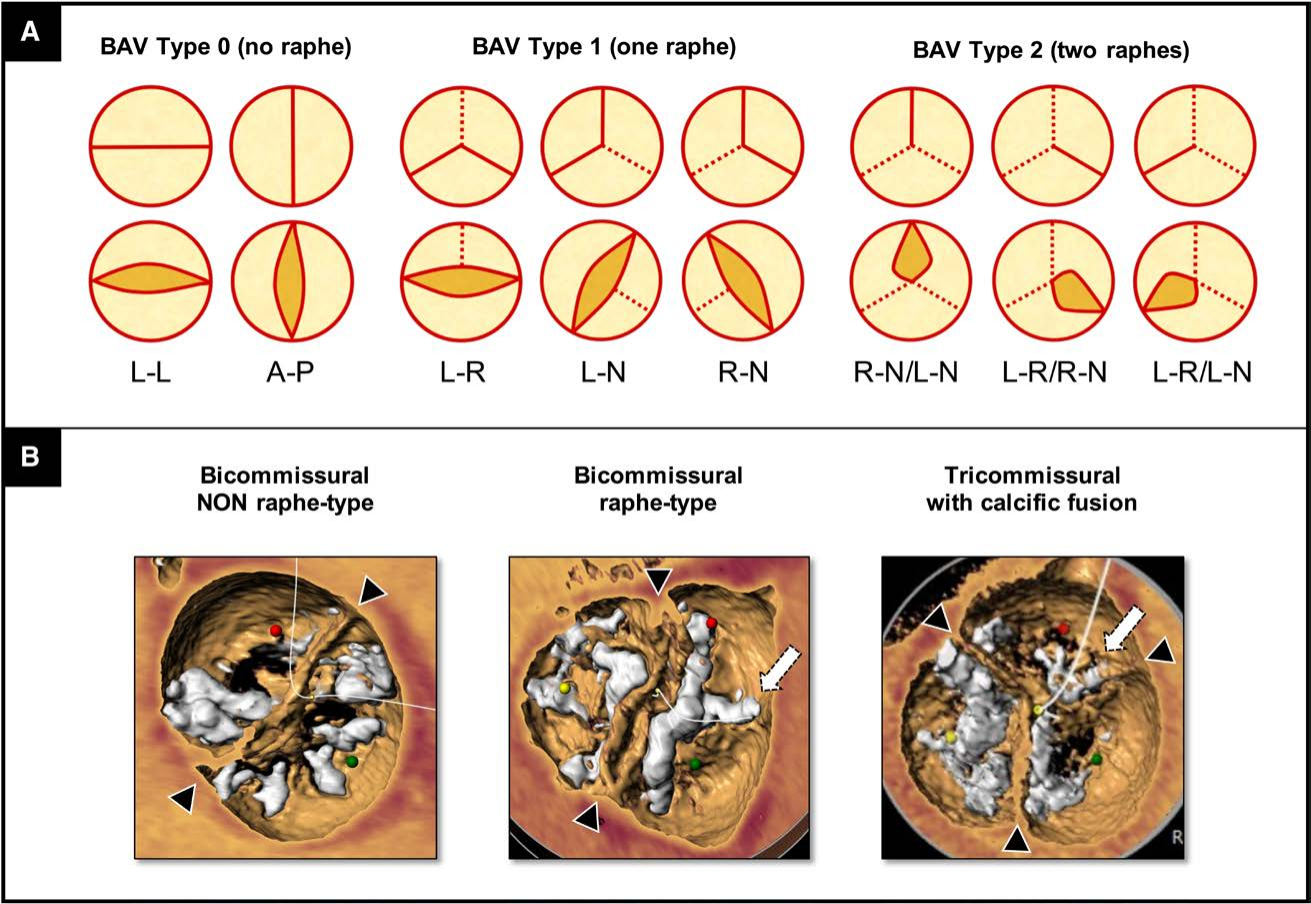
**Bicuspid aortic valve (BAV) classification systems.** **A**, Sievers and Schmidtke^8^ (surgical-derived) classification. Dotted line(s) denote(s) the raphe(s). **B**, Jilaihawi et al^4^ (computed tomography [CT]-derived) classification. Head arrow indicates the commissure. Arrow indicates the raphe or commissural fixation. A indicates anterior; L, lateral (in BAV type 0) or left (in BAV type 1 or 2); N, noncoronary; P, posterior; and R, right.

The characterization of BAV phenotypes by multislice computed tomography (CT) plays a key role in TAVR planning. As BAV includes a variety of morphological abnormalities (eg, undeveloped cusp, cusp fusion by raphe, acquired commissural fusion, etc),^1^ it is recommended to obtain a dynamic full cardiac cycle acquisition for accurate aortic valve evaluation. Different classification systems of BAV exist. The most widely used is that of Sievers and Schmidtke,^8^ which describes 3 types of BAV (0, 1, and 2) based on the number of raphes, with associated subcategories (Figure 2A). More recently, a TAVR-specific classification was suggested by Jilaihawi et al,^4^ depicting 3 BAV phenotypes based on the presence or absence of raphe and the number of commissures (Figure 2B): bicommissural nonraphe type (like Sievers type 0); bicommissural raphe-type (equivalent to Sievers type 1); and tricommissural BAV, a morphology with some anatomic features similar to Sievers type 1 and others similar to tricuspid aortic valve (TAV), often described as acquired (indeterminate Sievers class). This classification values the pathological substrate (presence of raphe and number of commissures) impacting the interaction between the THVs and BAV (Figure IA in the Data Supplement). In particular, the distinction between the bicommissural raphe type and tricommissural BAV has important operative functional implications, as a congenital raphe may oppose a higher resistance to adequate prosthesis expansion than an acquired commissural fusion (Figure IB in the Data Supplement). Outcomes of TAVR in bicuspid patients are strictly related to the BAV phenotype. Jilaihawi et al^4^ first reported higher rates of PVL and new permanent pacemaker implantation in bicommissural raphe-type and tricommissural BAV patients as compared to TAV group. More recently, a large-scale study showed that the presence or absence of severe calcification of both raphe and leaflets in raphe-type BAVs clearly stratify the outcomes with new-generation devices, in terms of increased risk of aortic injury, PVL, and overall mortality.^9^ Thus, the detection of high-risk BAV phenotypes should guide the decision-making process when evaluating TAVR for low surgical risk and younger bicuspid patients.

### Virtual Basal Ring (Annular) Evaluation

In tricuspid AS, the so-called virtual basal ring (VBR) is the tightest part of the aortic root, where the THV will anchor and seal.^10^ Hence, this virtual structure, bounded by 3 anatomic hinge points at the nadir of each of the aortic cusp insertions, represents the appropriate reference plane for prosthesis sizing and implantation height.^10^ However, in BAV, the anatomic definition of the VBR may be more difficult:

1. In BAV without raphe, the definition of a 3-dimensional structure (ie, the VBR) is challenging because of the presence of only 2 anatomic hinge points. Manual multislice CT assessment of annulus should be chosen over automatic detection since it is more accurate in alignment of the cusps (Figure II in the Data Supplement).^11^
2. In bicommissural raphe-type and in tricommissural BAV, the VBR definition is more accurate as 3 cusps are easily recognizable. Nevertheless, the detection of the cusps nadir might be difficult because of their typical unequal size (fused versus nonfused cusp).^1^

**Figure 3.**
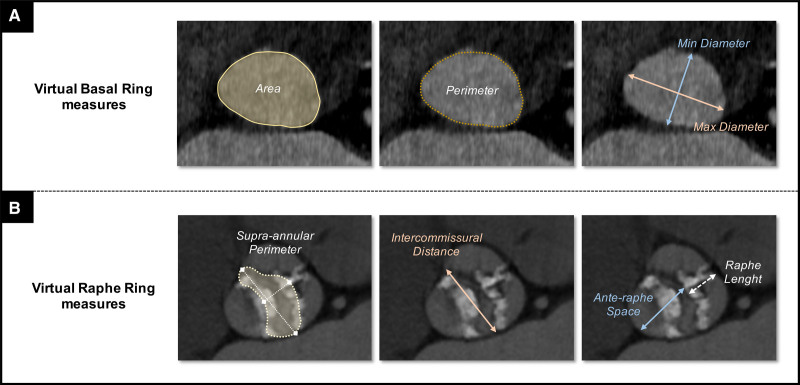
**Sizing strategies in bicuspid aortic valve.** **A**, Standard computed tomography (CT)-based measurements at virtual basal ring. **B**, Proposed CT-based measurements at virtual raphe ring.

Multislice CT-based measures of VBR should include both area and perimeter (with respective derived mean diameters) for standard annular prosthesis sizing, as well as major and minor diameters for annular eccentricity assessment (Figure 3A).

## Virtual Raphe Ring (Supraannular) Evaluation

Recently, some authors suggested a supraannular plane, the virtual raphe ring (VRR) in analogy with the VBR, for prosthesis sizing in BAV. In fact, in contrast to classical tricuspid AS, in stenotic BAV, the tightest part of the aortic root for THV anchoring and sealing might not be the traditional VBR but rather the raphe plane.^12^ However, this concept of supraannular sizing is still less standardized, leading to high variability across operators.^13–16^ The key issues when applying a VRR-based sizing strategy are what and where to measure.

With regard to the what, operators should not rely on a single parameter, but rather perform a multiparametric assessment considering (Figure 3B):

1. Supraannular perimeter: The projected neoannulus (ie, the VRR) can be traced drawing the internal leaflets margins with the valve open in systole. Due to the irregular shape of this supraannular structure, the perimeter (with derived diameter) rather than area should be calculated, possibly avoiding all the bulky surrounding structures (ie, calcific raphe and cusps calcification) that can reduce the space for THV expansion (see the example shown in Figure 3B). However, a definitive generalization of the supraannular perimeter measure cannot be formulated due to the high variability of supraannular anatomy itself.
2. Intercommissural distance (corresponding to the major supraannular diameter).
3. Ante-raphe space (corresponding to the minor supraannular diameter).
4. Raphe-specific measurements (length, degree, and distribution of calcification). A raphe length >50% of area- or perimeter-derived annulus diameter and a high calcium volume (>300 mm^3^) have been associated with low raphe compliance to prosthesis expansion.^15^

With regard to where to measure, the proper height to consider for the VRR plane is still a matter of debate. For some authors, the supraannular plane should be sited at 4 to 5 mm from the annular plane.^13,14^ However, the selection of an identical VRR height for all BAVs should be considered arbitrary, as the extension of commissural fusion is variable.^4^ Rather, it seems preferable to consider the plane where the supraannular structure has the tightest dimensions.^12^ In support of this, a post-TAVR CT scan study in raphe-type BAVs showed that the level of maximal THV constriction matches that of highest protrusion of raphe along the aortic root.^17^ In BAV nonraphe type, as the raphe plane does not exist by definition, a virtual commissural ring should be alternatively considered, with supraannular sizing limited to the perimeter of the valve orifice and to the intercommissural distance.

### Aortoventricular Complex Assessment

As in tricuspid AS, a comprehensive evaluation of the aortoventricular complex should include additional anatomic features, such as left ventricle outflow tract sizes and calcification, sinuses of Valsalva dimensions, coronary ostia height, and spatial relationship with respect to raphe orientation, sinotubular junction width, and ascending aorta diameter and angulation.^10,18^ Combining these data with the morphological features of BAV may help to select the most appropriate THV, as well as to foresee procedural pitfalls (Figure III in the Data Supplement).

### Prosthesis Size Selection

**Figure 4.**
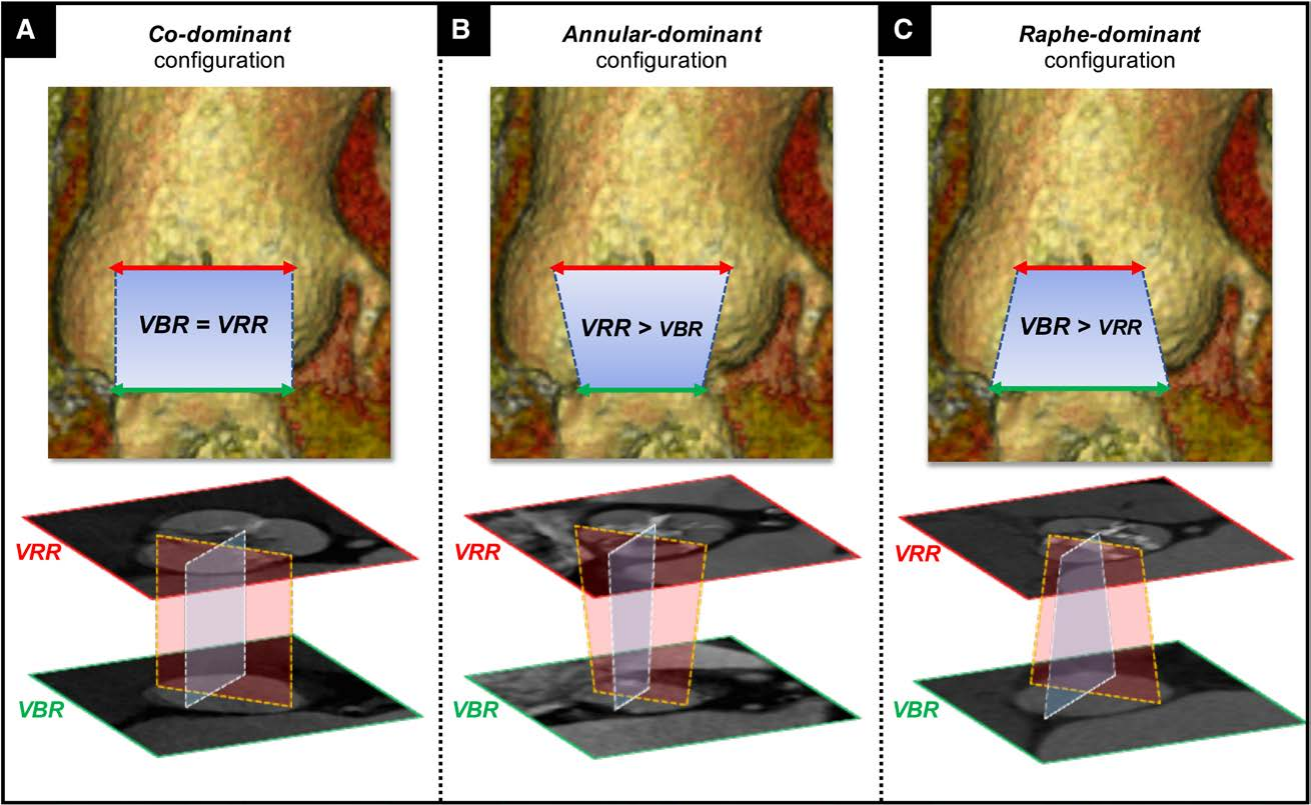
**Transcatheter heart valve (THV) sizing in bicuspid aortic valve (BAV).** Different patterns of landing zone in BAV. Codominant (**A**) and annular-dominant (**B**) with indicated standard THV annular-sizing strategy. Raphe-dominant (**C**) with advised THV supraannular sizing strategy.

Comparing the annular (VBR) and supraannular (VRR) sizes, 3 aortic patterns can be encountered (Figure 4)^13,14,16^: (1) Codominant, when VBR and VRR sizes are similar (Figure 4A); (2) Annular or VBR-dominant, when VBR is smaller than VRR (Figure 4B); and (3) Raphe or VRR-dominant, when VRR is smaller than VBR (Figure 4C). In the first 2 scenarios, a conventional annular-based THV sizing can be safely applied, whereas in the third pattern a supraannular sizing strategy (aimed at the VRR) should be considered in selected cases (eg, severely calcific raphe) to avoid significant under expansion of the (oversized if annular based) prosthesis.^9,19^

Notably, the prevalence of these 3 configurations varies according to the BAV type:

1. Bicommissural nonraphe type: Either a codominant or VBR-dominant pattern is almost equally found in this BAV type.^13,14^ Accordingly, annular-based THV sizing (ie, similar to tricuspid AS) is the most used, yielding to acceptable results with minimal valve oversizing.^13,14^
2. Bicommissural raphe-type: Although codominant and VBR-dominant patterns are the most frequently encountered, with annular-based sizing which remains the default approach, a VRR-dominant pattern may be found in up to 20% of cases.^13,14^ Thus, a VRR-based sizing strategy should be necessary in selected cases, leading to appropriate THV downsizing (with respect to annular sizing).^13–16^
3. Tricommissural BAV: THV sizing largely depends on length, degree and extent of calcification of the commissural fusion. The lower the compliance of raphe, the higher the likelihood for a VRR-dominant behavior. Notably, the proportion of a VRR-dominant shape with suggested THV downsizing was as high as 27.1% in this BAV anatomy.^14^

**Figure 5.**
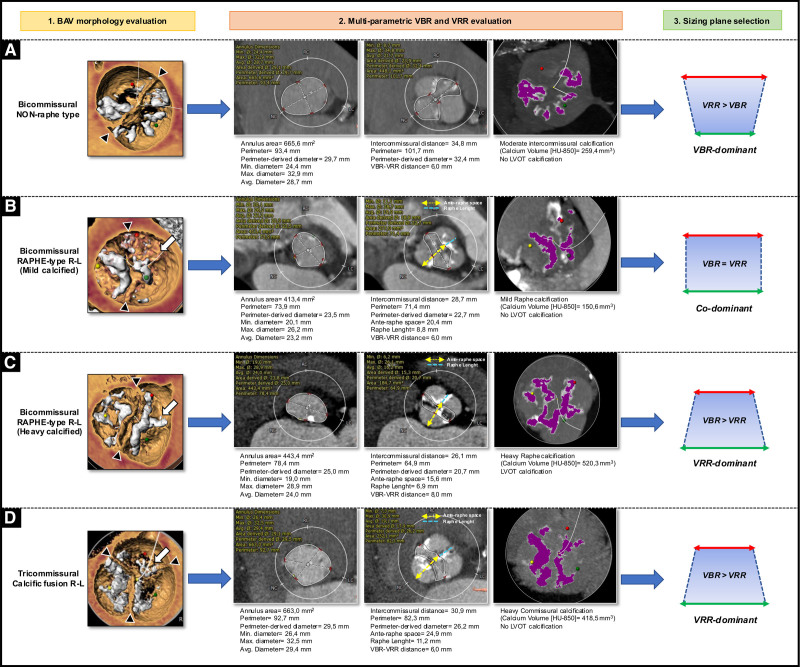
**Explicative cases of transcatheter heart valve (THV) sizing in bicuspid aortic valve (BAV).** THV sizing based on BAV phenotype (1) and multiparametric evaluation of virtual basal ring (VBR) and virtual raphe ring (VRR; 2). **A**, Bicommissural nonraphe type: final annular sizing (VBR-dominant pattern). **B**, Bicommissural raphe-type with R-L cusps fusion by mild-calcified raphe: final annular sizing (codominant pattern). **C**, Bicommissural raphe-type with R-L cusps fusion by severe-calcified raphe: final supraannular sizing (VRR-dominant pattern). **D**, Tricommissural BAV with calcific commissural fusion: final supraannular sizing (VRR-dominant pattern). Black head arrow indicates the commissure. White arrow indicates the raphe or commissural fusion. L indicates left; LVOT, left ventricular outflow tract; and R, right.

Figure 5 illustrates explicative cases of different approaches for THV sizing according to BAV phenotypes.

### Prosthesis Type Selection

As mentioned above, the introduction of the new-generation THVs improved the device success rate in BAV similarly to TAV.^3,5–7,9,13,14,16,19–29^ With regard to THV choice in bicuspid anatomy, in the BEAT registry (Balloon Versus Self-Expandable Valve for the Treatment of Bicuspid Aortic Valve Stenosis), the balloon-expandable Sapien 3 (Edwards Lifesciences) THV had higher residual gradients and a trend towards higher rates of annular rupture, while the self-expanding CoreValve Evolut R/PRO (Medtronic) valves had higher rates of moderate or severe PVL.^20^ Granted that, in the absence of randomized comparisons among different types of THVs in BAV (Table), a definitive recommendation on the preferable THV cannot be formulated. Rather, a patient-tailored device selection, based on different THV features, geometries, and interactions with the surrounding anatomy, is recommended. Figure IV in the Data Supplement summarizes the main features of new-generation THVs with regard to bicuspid AS treatment:

**Table.**
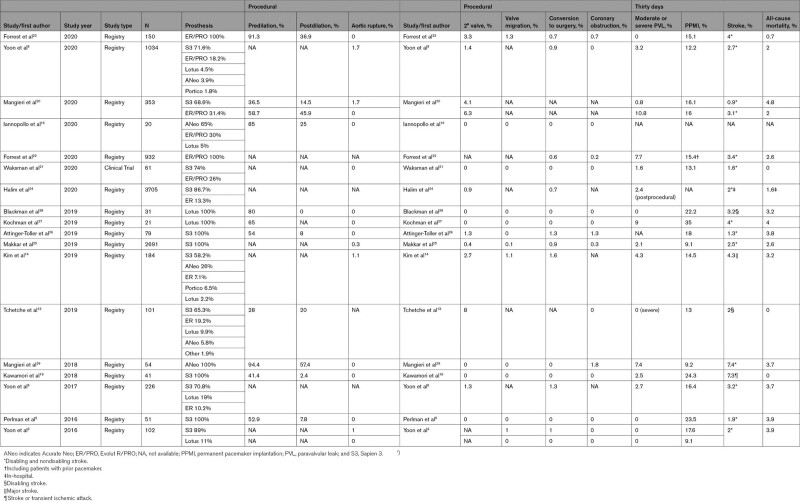
Outcomes of Patients With Bicuspid Aortic Valve Stenosis Treated With Current Generation of Transcatheter Heart Valves in Main Registries

1. Balloon-expandable THVs: The new-generation Sapien 3 valve is characterized by an external skirt that improves sealing in complex anatomies, such as irregularly shaped and calcific landing zone of BAV, reducing the need for oversizing. The high radial strength might theoretically prevent major valve underexpansion, as well as minimize the gap at the intercommissural triangles. This feature should be balanced with the risk of aortic injury in high-risk BAV phenotype or preexisting aortopathy. In the bicuspid arm of the Low-Risk TAVR trial,^21^ where 74% of patients received the Sapien 3 THV, zero mortality and no disabling strokes were reported at 30 days, with low rates of procedure-related complications, and excellent hemodynamic parameters.^21^ Data from the low-risk bicuspid group of the TVT Registry are forthcoming.
2. Self-expandable THVs: These devices might better adapt to the irregularly shaped landing zone of BAV. Moreover, the supraannular valve design may provide a better THV hemodynamic even in the presence of major under or asymmetrical expansion at the inflow portion.^13,22^ Finally, the repositioning and recapturing features of some of them might allow a more accurate positioning. The majority of evidence is available for the Evolut TAVR systems.^22,23^ In the recently published nonrandomized Low-Risk Bicuspid study,^23^ TAVR with the Evolut R/PRO THVs achieved favorable 30-day results, with low rates of death and stroke and high device success rate. The BIVOLUTX study (URL: https://www.clinicaltrials.gov; Unique identifier: NCT03495050) will explore outcomes and post-TAVR CT findings of BAV patients treated with the Evolut TAVR system.
3. Mechanically-expandable THVs: The unique mechanical expansion mechanism of the Lotus Edge (Boston Scientific) valve may reduce the risk of annular rupture.^27,28^ In addition to this, the device is fully repositionable and retrievable even when expanded, allowing optimal positioning even in unfavorable BAV anatomies. To note, the Lotus TAVR system was recently pulled off the market.

## Procedural Considerations

### Balloon Valvuloplasty/Sizing

Balloon aortic valvuloplasty can be used in addition to a multislice CT scan for THV sizing in selected cases, as it helps to unmask the mechanical characteristics of the annular and supraannular structures by mimicking the interaction with the THV. Specifically, balloon aortic predilation allows to (1) facilitate THV crossing, proper positioning, and expansion; (2) test raphe’s resistance, predicting THV expansion; (3) define device size for borderline/uncertain CT-based sizing; and (4) foresee the risk of residual leakage and coronary obstruction by supraannular aortography.

With regard to balloon sizing technique, a balloon indentation (waist sign) above the annulus, with less than mild contrast regurgitation, suggests that the supraannular structure may provide enough force for THV anchoring and could, therefore, be selected as the appropriate level for device sizing and implantation depth (VRR-dominant).^30^ Figure V in the Data Supplement illustrates explicative cases of balloon sizing use in TAVR for BAV.

### Prosthesis Implantation Technique

**Figure 6.**
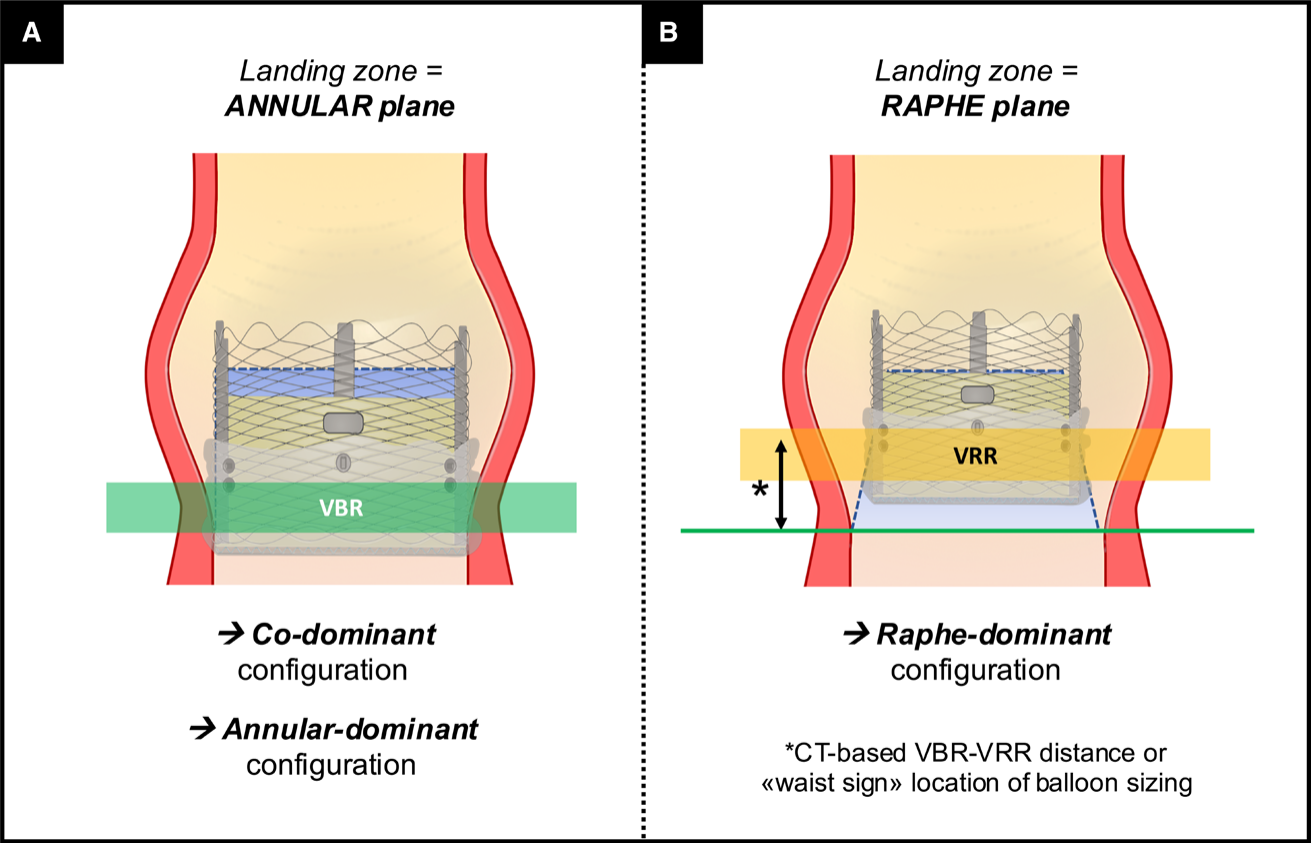
**Transcatheter heart valve (THV) positioning in bicuspid aortic valve.** Illustration showing standard THV implantation at virtual basal ring (VBR) in case of codominant or annular-dominant shapes (**A**), and optional higher THV implantation at virtual raphe ring (VRR) in case of raphe-dominant shape (**B**).

According to the 3 aortic root configurations for THV sizing, the operator can aim for one of 2 different landing zones (Figure 6): (1) in codominant and VBR-dominant scenarios, the VBR is the proper landing zone (Figure 6A) and (2) in VRR-dominant pattern, a supraannular landing zone is advisable based on raphe characteristics (Figure 6B). When the VRR is the selected landing zone, THV implantation height should be targeted for optimal supraannular sealing (Figure VI in the Data Supplement).^12,31^ Although a higher prosthesis implant may theoretically reduce the risk of conduction disorders (by reducing the interaction with the conduction pathway),^5^ this approach might carry some pitfalls (eg, device migration and coronary obstruction) based on aortic root anatomy. It is important to note that determining the optimal angiographic projection for THV deployment may be complex in BAV, as the leaflets appear irregular at fluoroscopy. Therefore, correct coaxial alignment of the THV frame should be based on the combination of CT-derived orthogonal view and orientation of the valve at fluoroscopy.

### Balloon Postdilatation

Although postdilatation might mitigate BAV-related THV distortion, it should be limited to those cases with residual significant PVL or transvalvular gradient due to the potential increase of balloon-related complications. A second view orthogonal to that of valve deployment is highly recommended to unmask possible stent frame underexpansion (Figure VII in the Data Supplement, upper), prompting the invasive evaluation of transprosthetic gradients. If postdilatation is required, the final balloon size should depend on balloon compliance, presence of adverse aortic root features (eg, calcific left ventricle outflow tract), and the smallest diameter at VBR or VRR level (Figure VII in the Data Supplement, lower). In the BEAT registry, the rate of postdilatation was significantly higher with the self-expandable Evolut R/PRO THVs compared with balloon-expandable Sapien 3 valve.^20^ The long-term impact of BAV-related THV distortion on structural valve degeneration, bioprostheses failure, and leaflet thrombosis is still to be fully evaluated.^4,13,19^

### Stroke Risk and Cerebral Embolic Protection

TAVR in BAV seems to be associated with a higher risk of stroke compared with tricuspid AS.^25^ This increased risk might be explained by the higher calcium burden of BAV and the higher number of maneuvers required during TAVR, such as the need for both balloon predilatation and postdilatation or the multiple attempts (in case of retrievable THVs) for valve positioning.^20^ The promising role of cerebral embolic protection devices during TAVR in BAV remains to be established.^23^

### Coronary Access

The reported rate of coronary obstruction after TAVR with current generation THVs in BAV patients is <2% (Table). Although BAV is usually associated with larger sinuses of Valsalva and higher coronary takeoff than TAV anatomy,^1^ borderline sinuses of Valsalva or coronary takeoff should raise more concerns to operators in BAV than in TAV, due to leaflet bulk. Moreover, some additional features require special attention. For instance, a calcific raphe located between the noncoronary and right coronary cusp might favor an asymmetrical displacement of the THV and leaflets towards the left coronary artery ostium. Furthermore, in BAV anatomy, the coronary ostia may be very close to the commissures (Figure III in the Data Supplement),^1^ leading to an increased risk of coronary obstruction, mostly when leaflets are long and bulky. Balloon aortic valvuloplasty with simultaneous aortography may help in confirming this risk. Advantages and pitfalls of coronary access (CA) after TAVR in BAV anatomy have been previously reported.^32^ In particular, when the coronary takeoff is high and the raphe is between the coronary cusps, the THV asymmetrical (ante-raphe) expansion may create a free space in front of the coronary ostia, which might facilitate the CA (Figure VIIIA through VIIIC in the Data Supplement).^32^ Contrastingly, when the THV is implanted higher up (Figure VIIID through VIIIF in the Data Supplement), CA after TAVR may be more challenging, mostly if the coronary takeoff is low or a commissural post randomly ends up in front of coronary ostia.^32^ The use of low frame profile THV or, alternatively, the commissural alignment technique for supraannular devices may increase the chance for selective coronary engagement.^33,34^ Closely related to this issue is the perspective of future need for valve-in-valve procedures. TAVR-in-TAVR procedure carries further challenges related to the risk of coronary obstruction and CA impairment. In fact, when the second THV is implanted, the leaflets of the first device are tilted up, creating a covered cage as high as the commissural posts.^35,36^ This aspect should guide the selection of the first THV in younger BAV subjects, as taller frame THVs increase the risk of both coronary obstruction and CA impairment after redo-TAVR.^35,36^ The BASILICA (Bioprosthetic Aortic Scallop Intentional Laceration to Prevent Iatrogenic Coronary Artery Obstruction) procedure to split the leaflets of native BAV (before TAVR) or THV (before TAVR-in-TAVR) might prevent the obstruction of coronary ostia and facilitate the CA.^37^ However, in the case of native BAV, the presence of leaflet calcium neighboring a calcified raphe may represents an important technical impediment to leaflet laceration.

## Limitations and Future Perspectives

Current evidence on TAVR for bicuspid AS is limited by the absence of randomized data and by important selection bias. In fact, younger and lower-risk patients, as well as those with large annulus or significant aortic dilatation, have been mostly excluded. Specifically designed randomized controlled TAVR trials both on bicuspid versus tricuspid AS and on balloon-expandable versus self-expandable THVs in BAV patients are needed. To date, the NOTION-2 (Nordic Aortic Valve Intervention; URL: https://www.clinicaltrials.gov; Unique identifier: NCT02825134) is the only randomized trial comparing TAVR versus surgical AVR for low-risk severe AS patients including BAVs.

## Conclusions

Bicuspid AS represents a complex anatomic scenario with unique challenges for TAVR and remains a surgical domain. Nevertheless, some patients with BAV may not be candidates for surgical AVR, and thus referred for TAVR. Advancements in preprocedural imaging, device iterations and procedural refinements will continue to improve the outcomes of these patients treated by TAVR. This review serves as a primer for those operators interested in expanding their practice in the percutaneous treatment of the highly variable anatomic setting of BAV.

## Sources of Funding

None.

## Disclosures

Dr Tarantini reports honoraria for lectures/consulting from Medtronic, Edwards Lifesciences, Boston Scientific, GADA, and Abbott. The other author reports no conflicts.

## Supplemental Materials

Online Figures I–VIII

## Supplementary Material
