# Supplementary material for: Transcatheter Aortic Valve Replacement for Bicuspid Aortic Valve Stenosis: A Practical Operative Overview
Source: Circ Cardiovasc Interv. 2021 Jun 16;14(7):e009827. doi: 10.1161/CIRCINTERVENTIONS.120.009827 (PMC10332651; doi:10.1161/CIRCINTERVENTIONS.120.009827)
Supplement: Supplementary file 1 [file hcv-14-e009827-s001.pdf]

SUPPLEMENTAL MATERIAL

Figure I. Multi-slice computed tomography (MSCT) imaging of bicuspid aortic valve (BAV) anatomy.

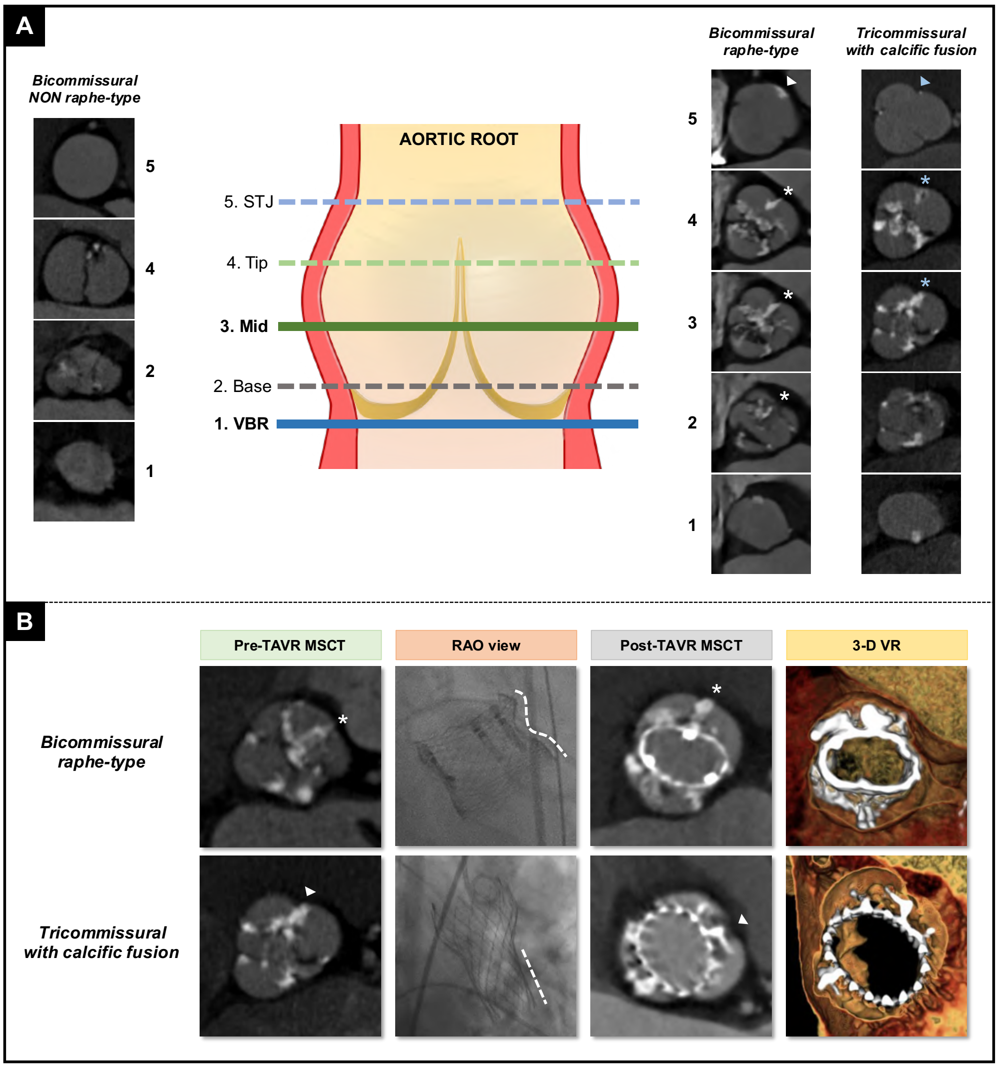

Panel A. MSCT analysis of the 3 TAVR-specific BAV morphologies at multiple cross-sectional levels: VBR (1), base of sinus (2), mid-sinus (3), upper margin of cusps (4), and STJ (5). In bicommissural raphe-type, the raphe (white asterisks) extents from the basal part of sinus for almost the entire cusp height, and the "non-opening" commissure does not reach the STJ (white head arrow); on the contrary, in tricommissural BAV type the acquired commissural fusion (blue asterisks) usually involves the upper portion of cusps, while the "non-opening" commissure reaches the STJ (blue head arrow).

Panel B. TAVR in bicommissural raphe-type (top) and in tricommissural BAV (bottom), with pre-procedural MSCT, procedural fluoroscopic RAO view, and post-TAVR MSCT images. Note that in tricommissural BAV with acquired commissural fusion the prosthesis shows better expansion with respect to bicommissural with congenital severe calcified raphe. Asterisk indicates the raphe. Head arrow indicates the acquired calcific commissural fusion.

TAVR indicates transcatheter aortic valve replacement; VBR, virtual basal ring; STJ, sino-tubular junction; and RAO, right anterior oblique.

Figure II. Multi-slice computed tomography-based annular sizing in bicuspid aortic valve (BAV) without raphe.

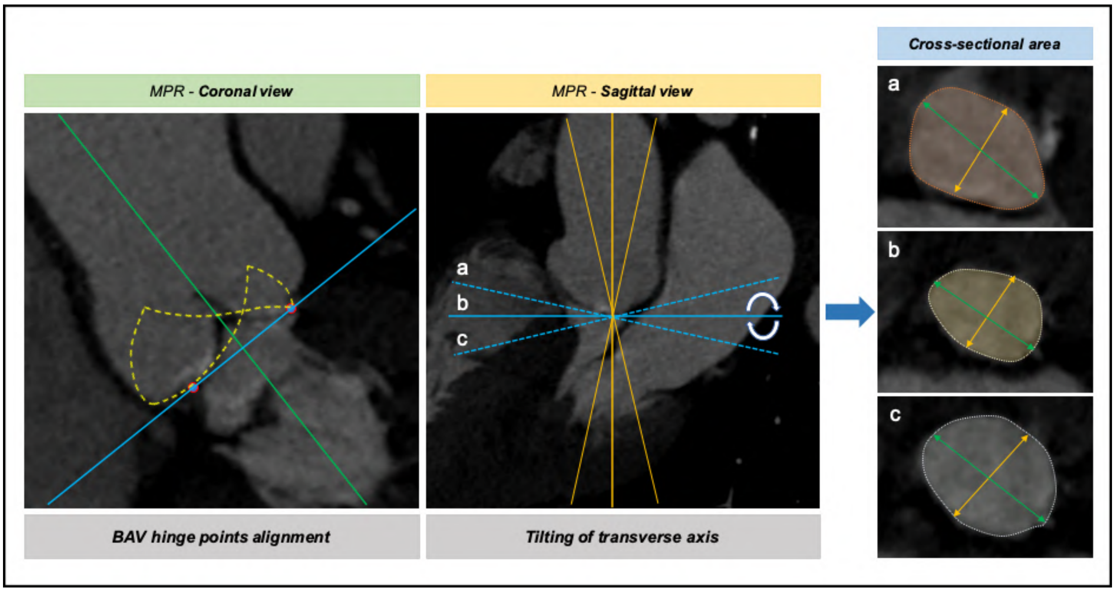

Illustration showing VBR identification in bicommissural non-raphe type. In contrast to tricuspid anatomy, a 3-dimensional structure (VBR) has to be reconstructed using only two anatomical hinge points (red points). After aligning the two most basal points of the cusps, attention should be paid in measuring the smallest cross-sectional area on the double-oblique transverse plane, as both under- and over-angulation may lead to overestimation of annular dimensions (right panels).

MPR indicates multi-planar reformation; and VBR, virtual basal ring.

Figure III. High-risk bicuspid aortic valve (BAV) features.

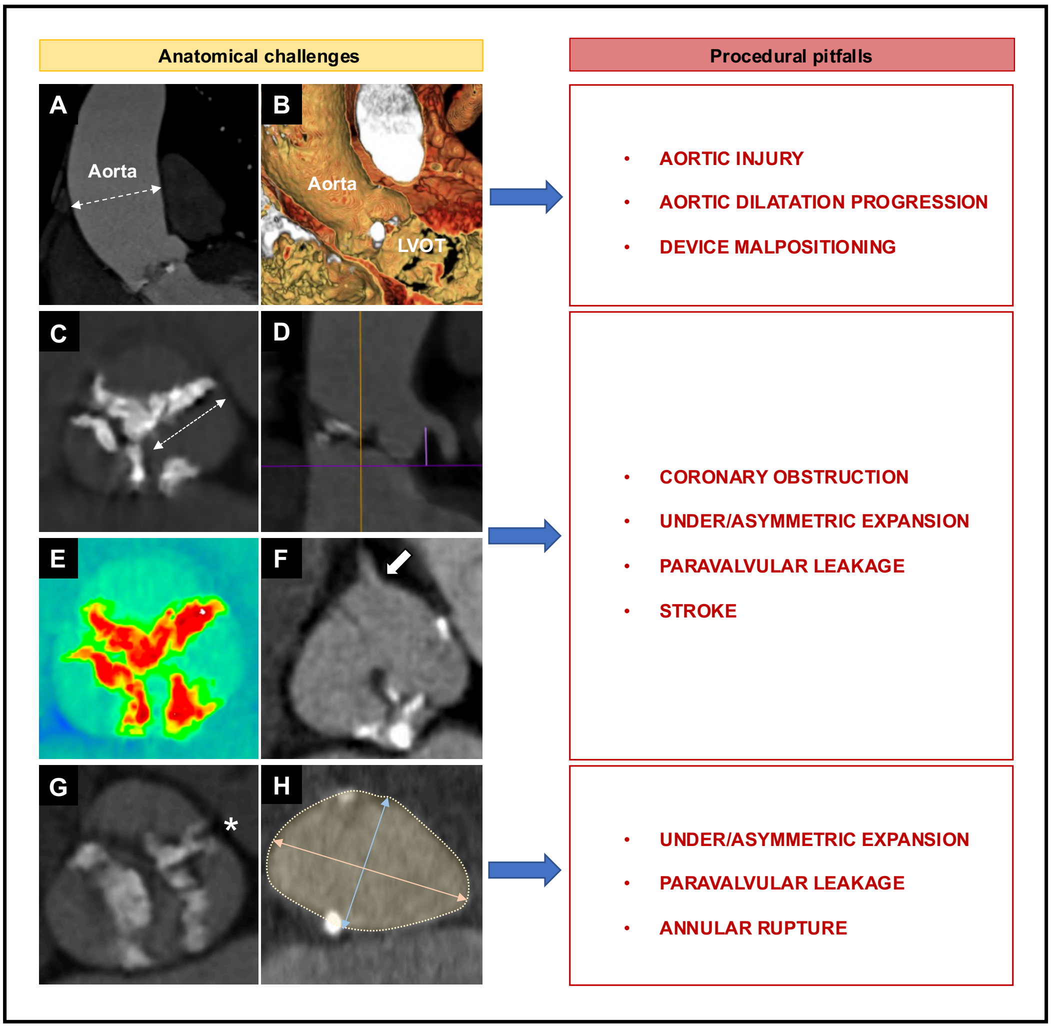

High-risk anatomical features associated with BAV anatomy that should be considered in procedural planning. Dilated (**A**) and horizontal (**B**) aorta; longer fused cusps (**C**); low coronary take-off (**D**); heavily calcified leaflets (**E**); coronary ostium origin proximal to the commissure (**F**); severe calcified raphe (**G**); pronounced oval-shaped annulus or calcification of left ventricle outflow tract (**H**).

Figure IV. New iterations of transcatheter heart valves (THVs) with relative pros and cons for bicuspid anatomy.

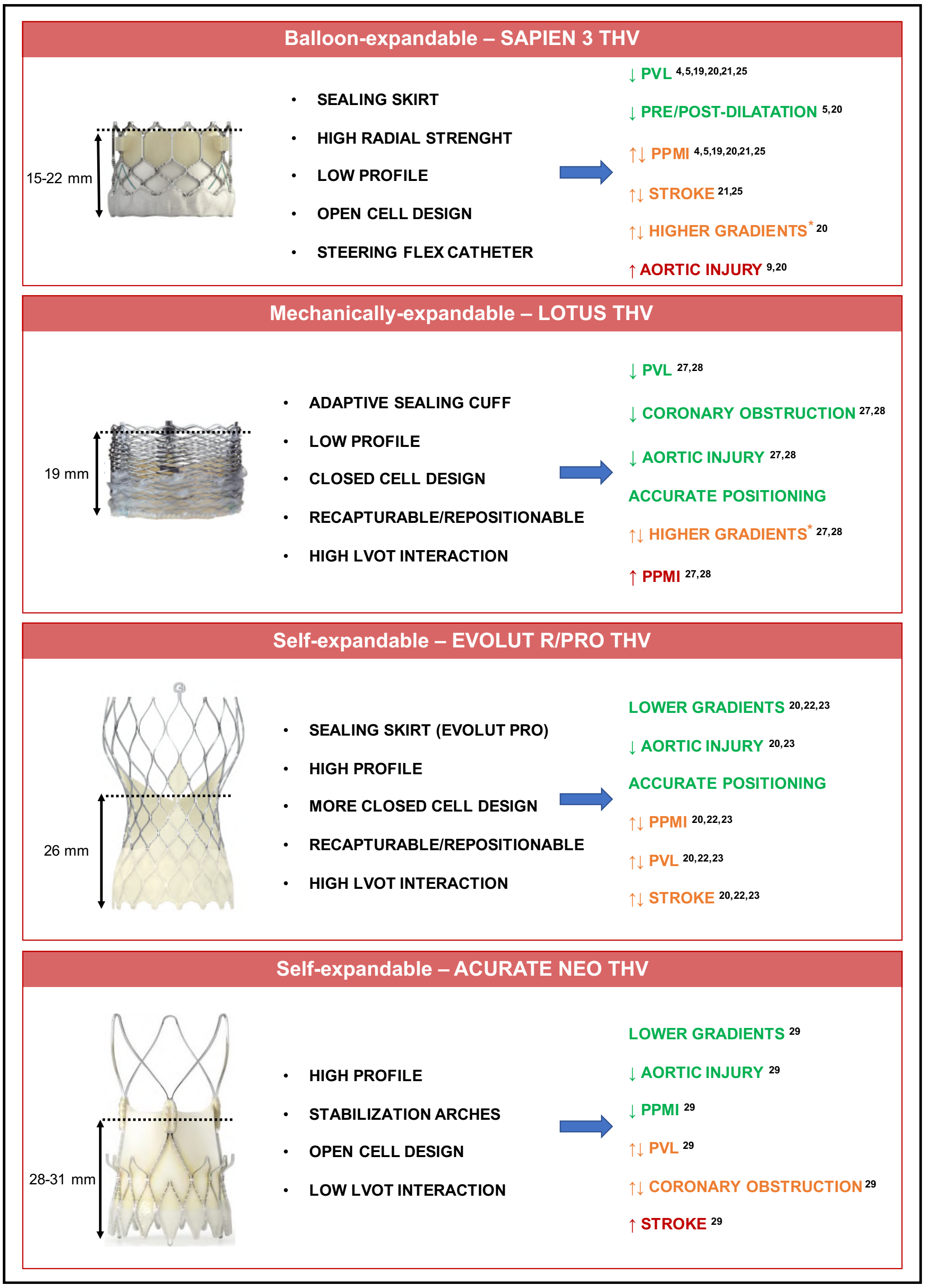

PVL indicates paravalvular leak; PPMI, permanent pacemaker implantation; and LVOT, left ventricle outflow tract. * for small valve sizes (Sapien 3 20/23 mm and Lotus 23 mm). See the main text for the cited references.

Figure V. Balloon sizing technique in bicuspid aortic valve.

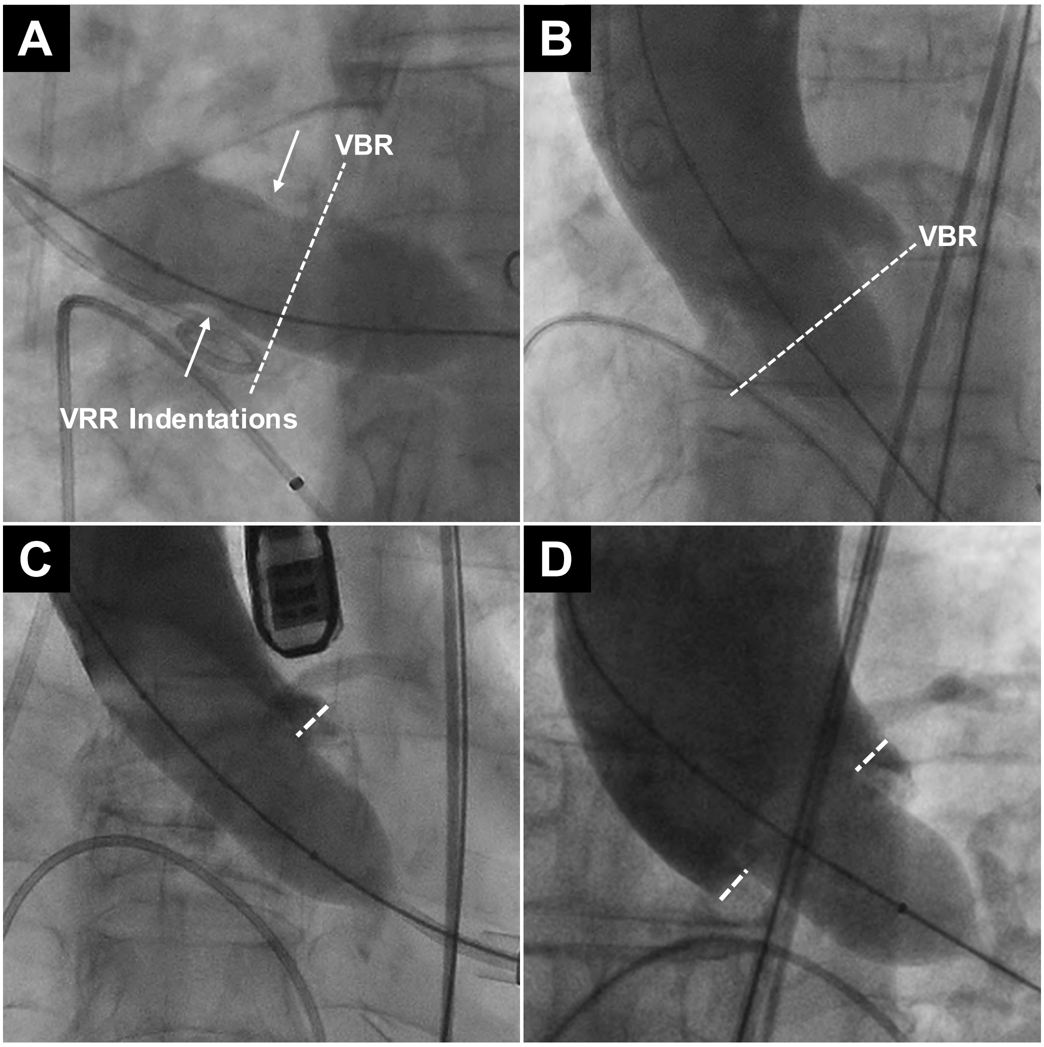

Supra-annular indentations of sizing balloon, suggesting a VRR-based sizing approach (**A**). Sizing balloon reaching the hinge points of VBR without supra-annular waist, suggesting a VBR-based sizing approach (**B**). Ante-raphe expansion of sizing balloon in bicommissural raphe-type, suggesting a low-compliance of the raphe (**C**). Symmetrical expansion of sizing balloon in bicommissural raphe-type, predicting full and symmetrical prosthesis expansion (**D**). Dotted line indicates the VBR.

VBR indicates virtual basal ring; and VRR, virtual raphe ring.

Figure VI. Transcatheter heart valve (THV) implantion height in bicuspid aortic valve (BAV).

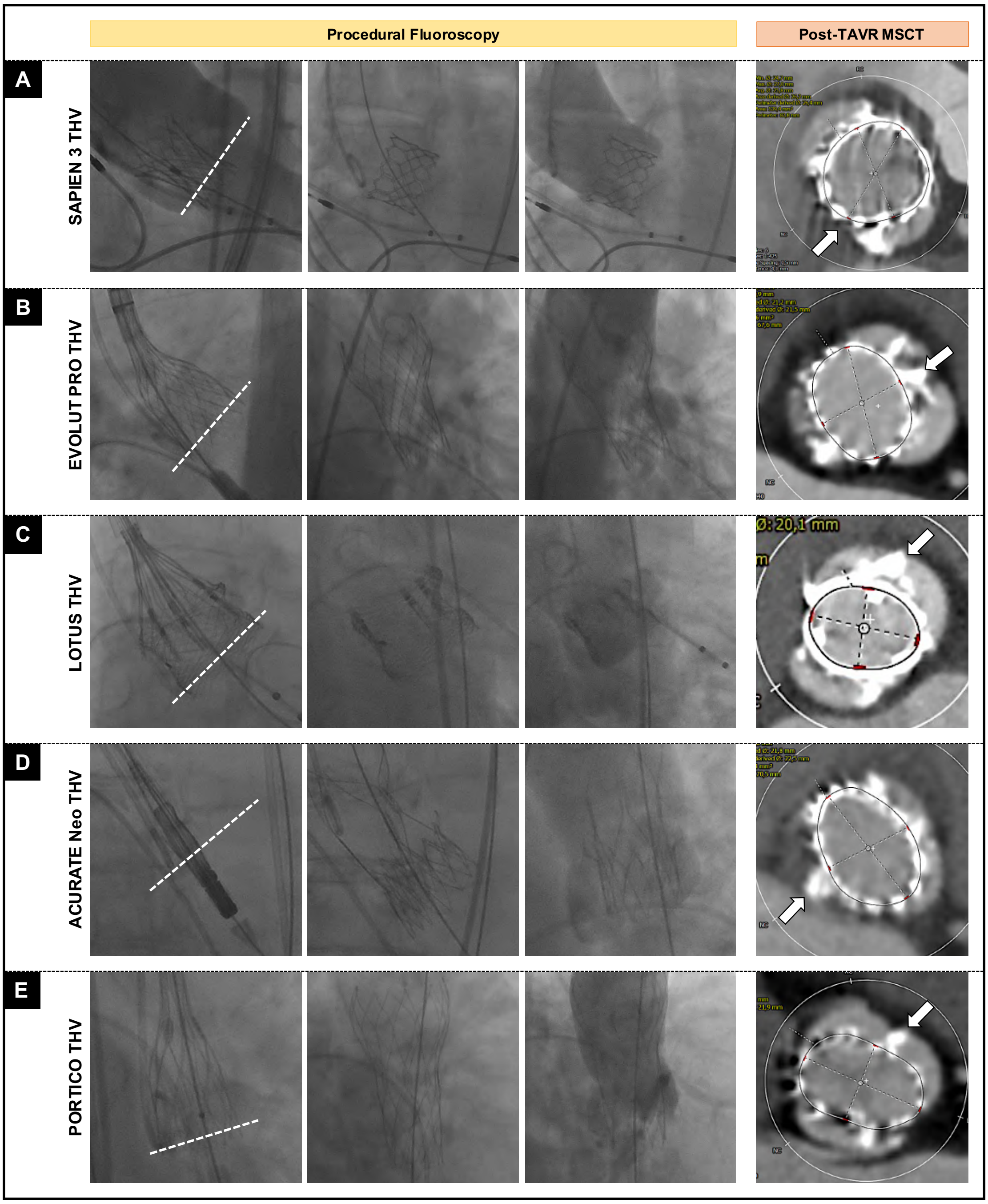

Explicative cases of final high THV implantation with optimal supra-annular sealing in BAV raphe-type with VRR-dominant pattern: Sapien 3 THV (**A**); CoreValve Evolut PRO THV (**B**); Lotus valve (**C**); Acurate Neo THV (**D**); Portico THV (**E**).

Dotted line indicates the plane of VBR. Arrow indicates the position of raphe.

VBR indicates virtual basal ring; VRR, virtual raphe ring; TAVR, transcatheter aortic valve replacement; and MSCT, multi-slice computed tomography.

Figure VII. Balloon post-dilatation in bicuspid aortic valve (BAV).

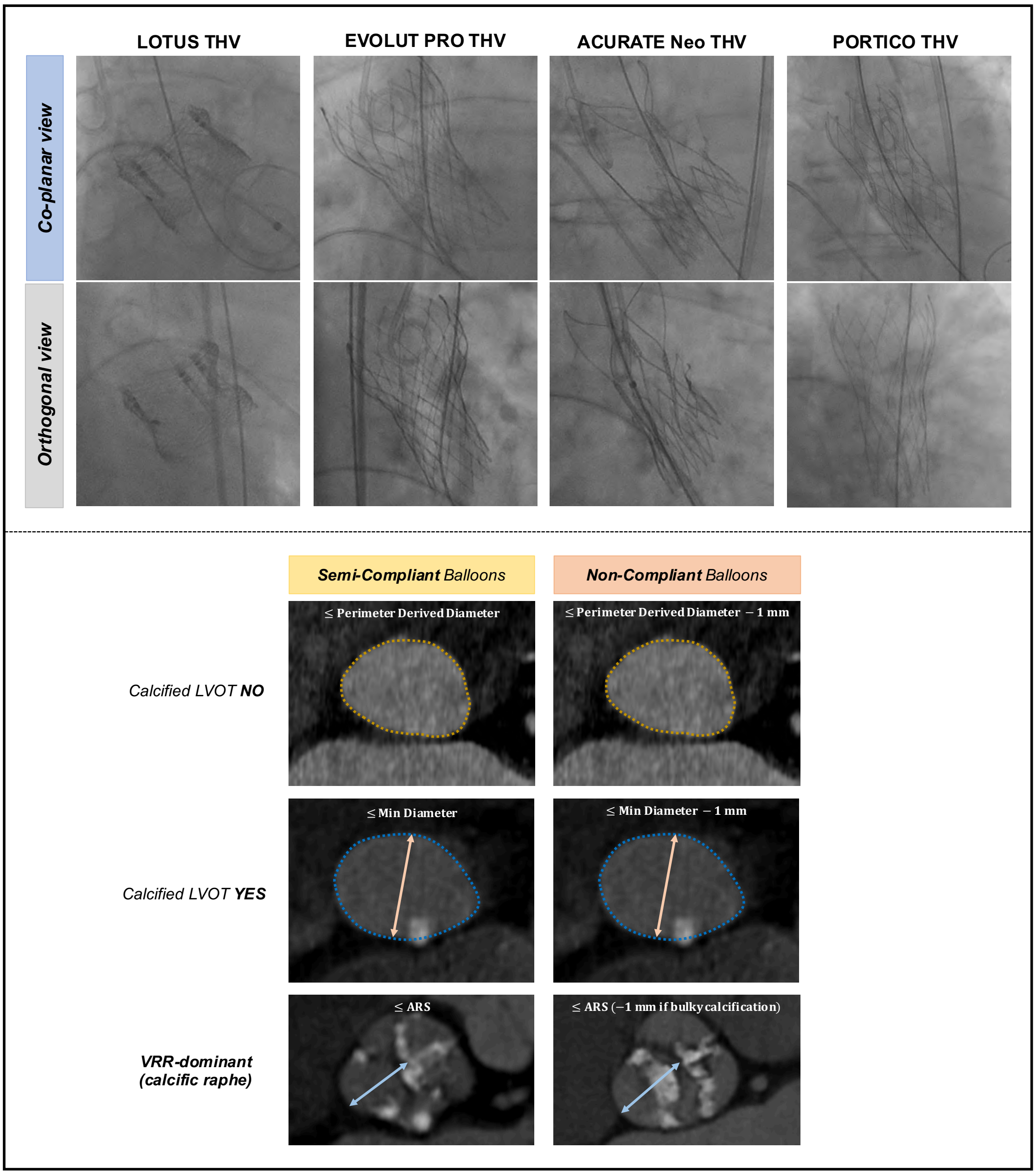

Upper Panel. Fluoroscopic images of different THVs implanted in bicommissural raphe-type BAVs. Note that obtaining an orthogonal view (preferably displaying the ante-raphe space by overlapping the fused cusps in raphe-type BAV) may help to unmask uneven expansion of the prosthesis stent frame, although what finally matters is trans-prosthetic gradients.

Lower Panel. Practical scheme to select balloon post-dilatation size in BAV, based on the balloon compliance, aortic root features, as well as the VBR and VRR sizes.

THV indicates transcatheter heart valve; VBR, virtual basal ring; VRR, virtual raphe ring; LVOT, left ventricle outflow tract; and ARS, ante-raphe space.

Figure VIII. Coronary access after transcatheter aortic valve replacement (TAVR) in bicuspid aortic valve (BAV).

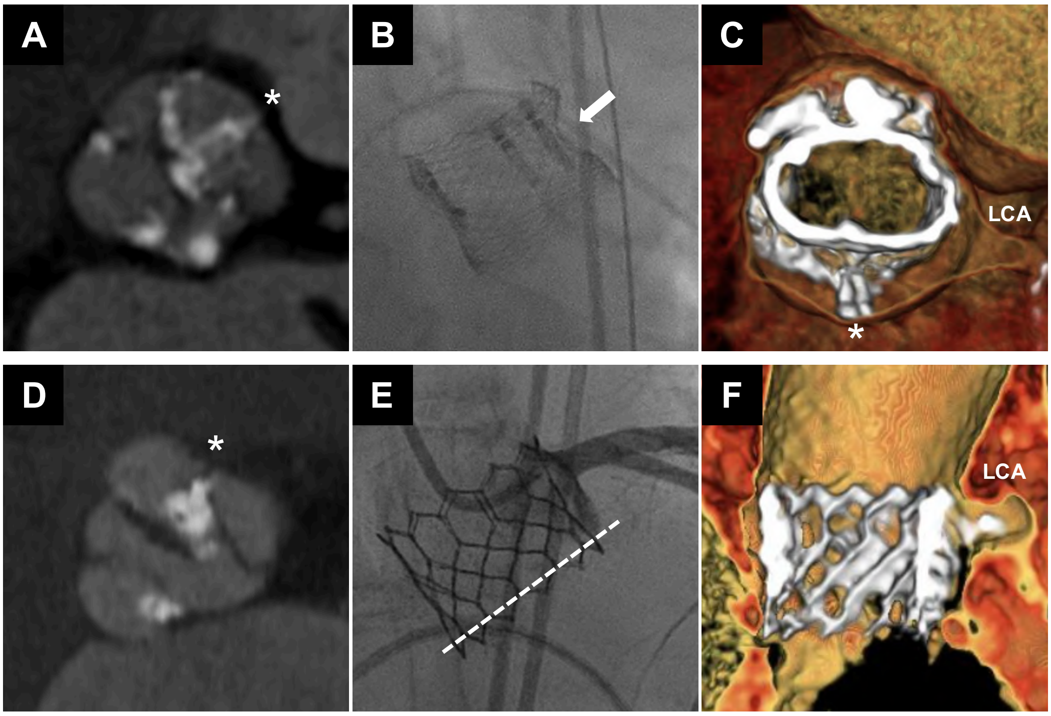

Pre-TAVR, procedural fluoroscopic, and post-TAVR images (from left to right) in 2 cases of bicommissural raphe-type BAV with right-left fusion (asterisks), which depict the 2 scenarios mentioned in the text (ante-raphe expansion and higher implantation) potentially impacting the coronary access after TAVR in BAV.

Panels A-C. Lotus THV sized and implanted according to standard annular (VBR) strategy, presenting an asymmetrical ante-raphe expansion due to the presence of a stiff heavily calcified raphe. Note the space between the asymmetrically expanded valve stent frame and the LCA ostium, which might facilitate the coronary cannulation in cases of low coronary origin and/or closed cell design of THV.

White arrow indicates the THV waist at the level of raphe.

Panels D-F. Sapien 3 THV sized and implanted according to supra-annular (VRR) strategy, resulting in higher implantation height (dotted line indicates the plane of VBR). To note the low profile and "open-cell" design of the Sapien 3 valve that may

permit easy incannulation of the LCA even though the coronary ostium lies below the upper margin of stent frame.

THV indicates transcatheter heart valve; VBR, virtual basal ring; VRR, virtual raphe ring; and LCA, left coronary artery.
